# Impact of cardiovascular disease on health-related quality of life among older adults in eastern China: evidence from a national cross-sectional survey

**DOI:** 10.3389/fpubh.2023.1300404

**Published:** 2024-01-15

**Authors:** Leping Wan, Guangmei Yang, Haiying Dong, Xiaoxiao Liang, Yan He

**Affiliations:** ^1^School of Management, Hainan Medical University, Hainan, China; ^2^School of Public Health and Emergency Management, Southern University of Science and Technology, Shenzhen, China; ^3^Department of Social Medicine and Health Care Management, School of Public Health, Zhengzhou University, Hainan, China

**Keywords:** health-related quality of life, cardiovascular disease, EQ-5D-3L scale, older adults, Eastern China

## Abstract

**Objective:**

This study explores the health-related quality of life (HRQoL) scores of Chinese older adults with Cardiovascular Disease(CVD) using the EQ-5D-3L, the aim of this study is to investigate the association between health and HRQoL in older adults with CVD.

**Methods:**

The data for this study were obtained from a cross-sectional study involving older adults residing in Chinese communities The EQ-5D-3L is used to measure the HRQoL scores in the older adults with CVD. One-way analyses were conducted using the Wilcoxon rank sum test and the Kruskal–Wallis H test to assess differences between groups. A binary logistic regression model was employed to analyze the influence each variable has on the presence of “any problem” on each dimension of EQ-5D-3L in older adults with CVD. An ordinal least squares (OLS) model is used to assess the relationship between older adults with CVD and HRQoL.

**Results:**

The mean EQ-5D-3L score for older adults with CVD is 0.774. 40.0% of older adults with CVD reported problems with pain/discomfort, followed by Mobility (35.9%), Self-care (31.5%), and Anxiety/depression (17.0%). Binary logistic regression models show that financial resources were the main factor influencing the five dimensions of EQ-5D-3L. The OLS model further indicates that younger age, financial resources, and a lower number of chronic conditions among older adults with CVD are associated with higher HRQoL scores.

**Conclusion:**

Chinese older adults with CVD have low HRQoL scores. Variousfactors influence both overall HRQoL scores and scores on each EQ-5D-3L dimension. This study is helpful in enhancing society’s attention to the HRQoL of older adults with CVD and taking targeted measures to improve them.

## 1 Introduction

Cardiovascular diseases (CVD), is a group of disorders that affect the heart and/or blood vessels and is now the leading cause of death, contributing to one-third of global deaths per year (1, 2). Growing evidence confirms that CVD incidences increase cardiovascular and all-cause mortality, especially among the older adults (3). In the 2019 American Heart Association data, the prevalence of cardiovascular disease averaged 75–78% in older adults with CVD aged 60–80 years and more than 85% in older adults with CVD aged 80 years and older (4). Some studies have indicated that gender significantly affects CVD incidence, in generally, before menopause, women are relatively protected from cardiovascular disease, and then, after menopause, the risk for cardiac disease greatly increases in women, leading to gender differences in outcomes between men and women attributable in large part to sex hormones and their associated receptors (5, 6). The world’s older adults continues to grow at an unprecedented rate. According to China’s 7th Census, 18.7% of people (617 million) in China are 60 years and older (7), and it is estimated that 4 million deaths are due to cardiovascular diseases each year in China (8). CVD and its treatments pose significant burden on patients’ health-related quality of life (HRQoL) and can affect their ability to function.

HRQoL is often used to assess the health status of patients, reflecting the patient’s physical, psychological, social, and emotional well-being (1). Moreover, HRQoL is considered an important patient-reported outcome measure for interventions and treatments in patients with CVD (9). With the public’s interest in HRQoL, more and more leaders are applying HRQoL to clinical care, preventive care, and health economic evaluation (10). The levels and determinants of HRQoL in the general population have been well documented in developed and developing countries (11–14). Several generic and disease-specific tools have been developed for measuring HRQoL of patients with CVD (15, 16). EQ-5D is a handy, easy-to-use tool for measuring health outcomes. The validity and reliability of EQ-5D have been tested in populations in mainland China (17, 18). And the EQ-5D-3L instrument has been validated in Chinese people and is being increasingly used for assessing HRQoL in people living with chronic conditions (19–22).

HRQoL is considered an important patient-reported outcome measure for interventions and treatments in patients with CVD (14, 21, 23). Although some previous studies also have reported the association between CVD and HRQoL; however, less is known about the association between cardiovascular health and HRQOL (24, 25). Accordingly, the aim of this study was to investigate the association between health and HRQoL in older adults with CVD. These findings may help provide a rationale for developing targeted interventions for older adults to improve patient health outcomes.

## 2 Method

### 2.1 Data collection

In this study, the eastern region is chosen as the target of the study. This cross-sectional investigation was conducted from September 2021 to December 2021 by the public health staff in the household survey. A multi-stage stratified sampling method was selected for this study. In the first stage, three provinces were randomly selected from 12 provinces in the eastern region, namely Shandong Province, Jiangsu Province and Guangdong Province. Secondly, one municipality was randomly selected from within each province. Qingdao in Shandong Province, Suzhou in Jiangsu Province and Guangzhou in Guangdong Province. In the second stage, a county/district was randomly selected within the jurisdiction of each city. Qingdao was chosen as Jimo District, Suzhou was chosen as Kunshan County and Guangzhou was chosen as Yuexiu District. Next, two streets were randomly selected from each city/district. Finally, two communities were randomly selected from each street. In the third stage, researches were randomly selected from each community.

**Figure 1:**
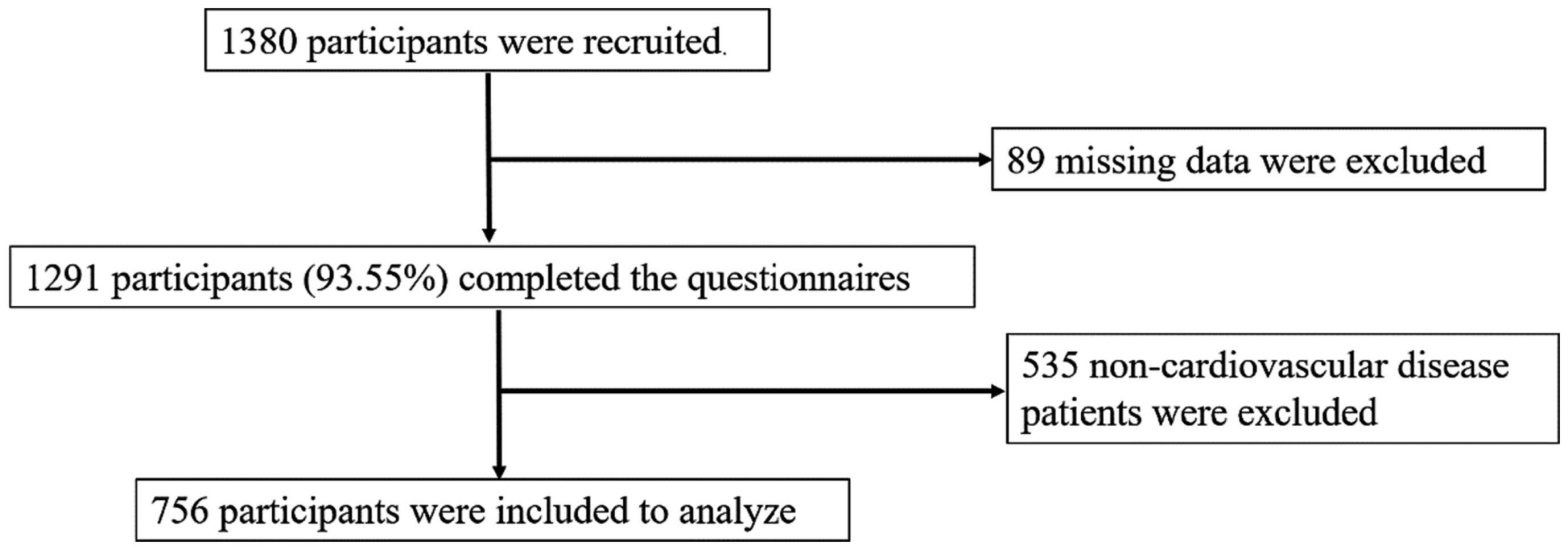
Flowchart on participant recruitment.

Inclusion criteria: (1) age ≥ 60 years, (2) receiving community-based home services for 6 months or more, (3) being conscious and able to communicate normally, (4) voluntarily participating in this survey. Exclusion criterion: (1) severe cognitive impairment, (2) severe memory impairment. Therefore, 1,380 questionnaires were collected, missing values of key variables were excluded from the data, and 1,291 valid questionnaires were recovered. Older adults with CVD were screened out, and 756 older adults were finally included. The recruitment process of participants is shown in Figure 1.

### 2.2 HRQoL score using EQ-5D-3L

The EQ-5D-3L was used to report the health status and evaluate the HRQoL score of older adults with CVD. The EQ-5D is the most commonly used quality of life scale in health research and as a generic preference based measure of HRQoL, it can be validly applied across participant groups and intervention types. The EQ-5D has been extensively studied and has been shown to have excellent reliability and validity, while the new 5 level version (as used in the current study) has been shown to reduce ceiling effects seen in people with minor health related quality of life impairments.

EQ-5D-3L has five dimensions: Mobility (MO), Self-care (SC), Usual Activities (UA), Pain/Discomfort (PD), and Anxiety/Depression (AD) Each dimension has three levels: no problem, some problems, and extreme problems.

Liu et al. (26) used the TTO method to translate the EQ-5D-3L measurements into scores. The EQ-5D-3L theoretically produces 243 health states (27). The EQ-5D-3L utility scores can be calculated according to Table 1, where C indicates a constant; MO2, SC2, UA2, PD2, and AD2 represent level 2 problems in mobility, self-care, usual activities, pain/discomfort, and anxiety/depression.; MO3, SC3, UA3, PD3, and AD3 represent the presence of level 3 problems in five dimensions of the scale, and N3 indicates that at least one dimension is at level 3. For example, the utility score of 32,213 = 1 − (0.039 + 0.246 + 0.105 + 0.074 + 0 + 0.205 + 0.022). The range of values is [−0.149 to 1]. The upper limit of effectiveness value is 1, which means that the state of full health is 11,111, and the lower limit is −0.149, which means the worst state of health is 33,333.

**Table 1:** Chinese utility values of EQ-5D-3L health status.

C	MO2	MO3	SC2	SC3	UA2	UA3	PD2	PD3	AD2	AD3	N3
0.039	0.099	0.246	0.105	0.208	0.074	0.193	0.092	0.236	0.086	0.205	0.022

### 2.3 Statistical analysis

Descriptive statistics were calculated for all measures. Means and standard deviations (SD) were presented for continuous variables, while frequency and percentages were used for categorical variables. A chi-squared test was used to compare the differences in the reported problems on each dimension of EQ-5D-3L across different groups. Since the EQ-5D-3L index scores were not normally distributed (Shapiro–Wilk test, *p* < 0.05). One-way analyses were performed using the Wilcoxon rank sum test and the Kruskal–Wallis H test to assess the differences in EQ-5D-3L scores between groups. A binary logistic regression model was used to predict the probability of participants reporting full health (0 and 1, where 0 indicates no problem and 1 indicates any problem reported) on each of the three dimensions of EQ-5D-3L. The ordinal least squares (OLS) model was used to explore the relationship between CVD and EQ-5D-3L score. Because ceiling effects are a common problem for EQ-5D-3L scores, we also performed Tobit regression analyses, which were consistent with those of OLS (see Supplementary Table S1).

## 3 Results

### 3.1 Participants’ characteristics

Females constituted 55.8% of the cohort, 33.0% were aged 60–70 years and 25.5% were 70–80 years of age. More women (23.1%) than men (15.7%) were above 80 years of age and also more women (14.2%) than men (12.5%) were between 70 and 79 years of age. The largest percentages of other variables were elementary school education (31.9%), income <$1,000 (34.5%), have three or more chronic diseases (48.1%), and sleep for 6–8 h (21.3%). See Table 2.

**Table 2:** The EQ-5D-3L score of the older adults with CVD (stratified by gender).

Variable	Total			Male		Female	
	*n* (%)	Mean (SD)	*p*	Mean (SD)	*p*	Mean (SD)	*p*
Age(years)			<0.001		<0.001		<0.001
60–70	263 (34.4)	0.875 (0.207)		0.842 (0.243)		0.904 (0.164)	
70–80	205 (26.8)	0.797 (0.247)		0.771 (0.274)		0.820 (0.219)	
>80	297 (38.8)	0.669 (0.295)		0.696 (0.298)		0.650 (0.293)	
Spouse			<0.001		<0.001		<0.001
Yes	490 (64.1)	0.825 (0.241)		0.812 (0.261)		0.834 (0.223)	
No	275 (35.9)	0.684 (0.294)		0.692 (0.295)		0.678 (0.294)	
Education			<0.001		0.003		<0.001
Illiteracy	223 (29.2)	0.694 (0.315)		0.694 (0.335)		0.64 (0.309)	
Primary school	244 (31.9)	0.775 (0.267)		0.730 (0.290)		0.816 (0.237)	
Junior high school	152 (19.9)	0.840 (0.212)		0.830 (0.235)		0.852 (0.182)	
High school and above	146 (19.1)	0.827 (0.217)		0.827 (0.233)		0.826 (0.200)	
Financial resources			<0.001		0.003		<0.001
Pension	418 (54.6)	0.796 (0.259)		0.780 (0.280)		0.810 (0.238)	
Support from family and friends	189 (24.7)	0.680 (0.308)		0.679 (0.311)		0.681 (0.308)	
Others	158 (20.7)	0.828 (0.218)		0.831 (0.217)		0.825 (0.219)	
Current living status			<0.001		<0.001		<0.001
Live alone	180 (23.5)	0.744 (0.268)		0.796 (0.240)		0.716 (0.279)	
Live with family	453 (59.2)	0.834 (0.225)		0.829 (0.238)		0.838 (0.216)	
Others	132 (17.3)	0.608 (0.331)		0.610 (0.333)		0.606 (0.239)	
Monthly income (￥)			0.117		0.115		0.384
<1,000	264 (34.5)	0.751 (0.283)		0.721 (0.299)		0.768 (0.273)	
1,001–3,000	198 (25.9)	0.806 (0.275)		0.824 (0.275)		0.795 (0.276)	
3,001–5,000	194 (25.4)	0.761 (0.248)		0.772 (0.245)		0.751 (0.251)	
>5,001	109 (14.2)	0.795 (0.260)		0.776 (0.292)		0.824 (0.198)	
Number of chronic diseases			<0.001		<0.001		<0.001
1	172 (22.5)	0.869 (0.223)		0.879 (0.218)		0.860 (0.229)	
2	225 (29.4)	0.808 (0.260)		0.818 (0.251)		0.800 (0.266)	
≥3	368 (48.1)	0.709 (0.279)		0.691 (0.297)		0.724 (0.263)	
Smoking			0.941		0.806		0.899
Yes	553 (72.3)	0.774 (0.275)		0.766 (0.293)		0.777 (0.268)	
No	212 (27.7)	0.775 (0.255)		0.774 (0.266)		0.783 (0.196)	
Alcohol			0.001		0.002		0.105
Yes	584 (76.3)	0.757 (0.284)		0.730 (0.309)		0.770 (0.269)	
No	181 (23.7)	0.830 (0.209)		0.827 (0.218)		0.840 (0.178)	
Sleep schedule (h)			0.001		0.018		0.032
<6	246 (32.2)	0.775 (0.256)		0.770 (0.281)		0.782 (0.240)	
6–8	356 (46.5)	0.803 (0.244)		0.807 (0.238)		0.801 (0.249)	
>8	163 (21.3)	0.705 (0.327)		0.701 (0.332)		0.709 (0.323)	

### 3.2 The EQ-5D-3L scores of older adults with CVD by gender

The HRQoL among older adults with CVD using the EQ-5D-3L was 0.774. 42.4% of older adults with CVD have a health index = 1 and are in perfect health. Females have a slightly higher health score (0.777) than males (0.770). Male and female older adults with CVD, who are younger, have a spouse, are highly educated, are financially independent (pension and other sources of income), live with their families, have a low number of chronic diseases, do not drink alcohol, and have a moderate amount of sleep, had higher HRQoL scores. See Table 2.

**Figure 2:**
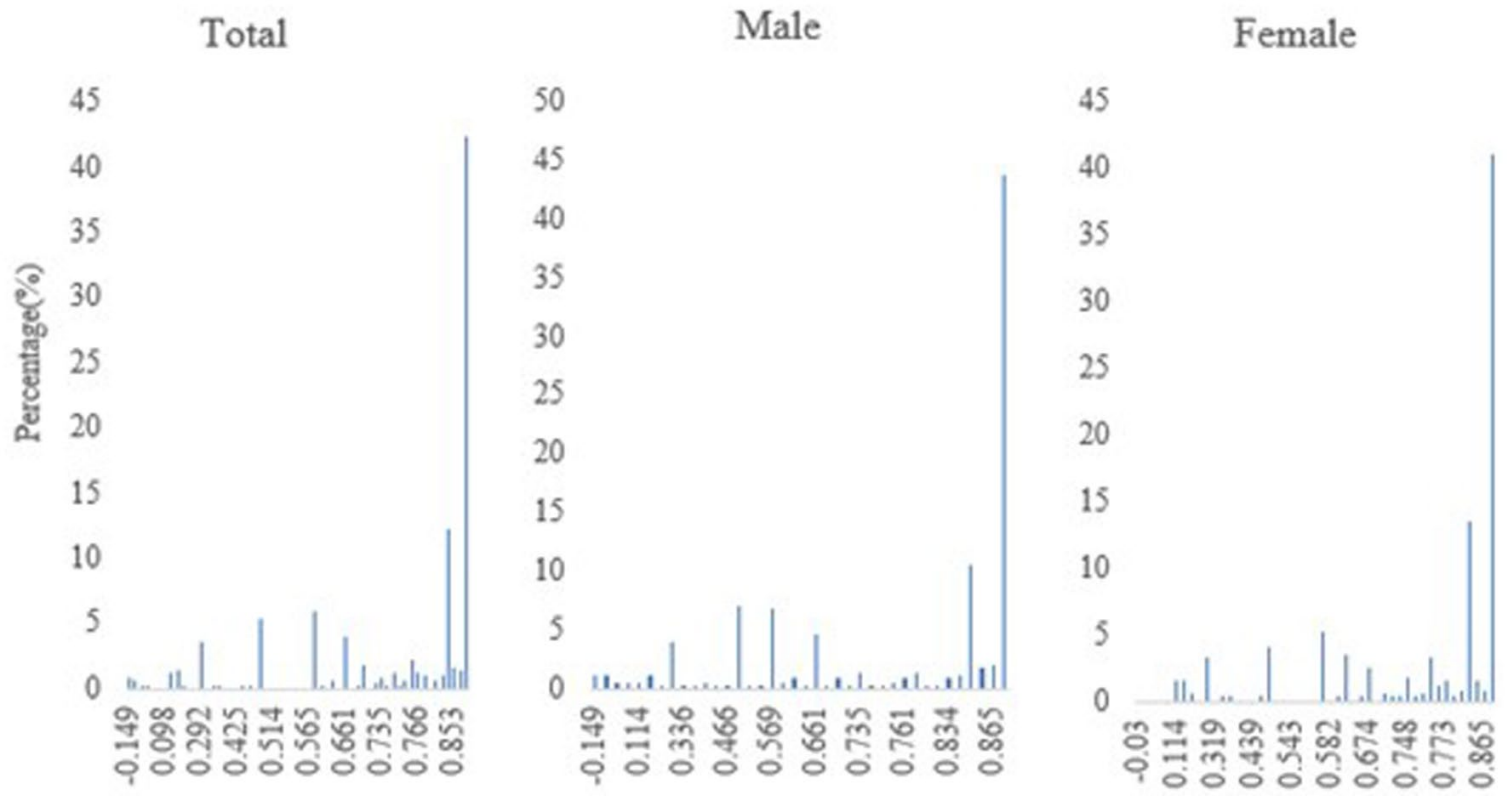
The distribution of EQ-5D-3L scores for total, and stratified by male and female.

The distribution of HRQoL scores by gender and for all older adults with CVD is shown in Figure 2.

### 3.3 Proportion of the five dimensions of EQ-5D-3L with any problems

Table 3 shows the percentage of the any problems response for each dimension of the EQ-5D-3L. The study results show that the five dimensions of PD, UA, MO, SC, and AD are problematic at 40.0, 36.2, 35.9, 31.5, and 17.0%, respectively. The proportion of older adults with CVD with the any problem response in each dimension is lower for those who are younger, financially independent (pension and other sources of income), and have fewer types of chronic diseases than the other groups. Older adults with CVD with a spouse, high literacy, and non-drinking behavior have lower proportions of the any problem response in the PD, MO, and UA dimensions than other groups. The proportion of older adults with CVD living with family members who had “any problem” in the PD, MO, UA, and SC dimensions was lower than in the other groups. Older adults with CVD with a monthly income of ￥3,001–5,000 have a higher proportion of the any problems response in the PD, MO, UA, and AD dimensions than any other group.

**Table 3:** Proportion of EQ-5D-3L reporting any problems in five dimensions.

Variable	Mobility		Self-care		Usual activities		Pain/discomfort		Anxiety/depression	
	*n* (%)	*p*	*n* (%)	*p*	*n* (%)	*p*	*n* (%)	*p*	*n* (%)	*p*
Overall	275 (35.9)		241 (31.5)		277 (36.2)		306 (40.0)		130 (17.0)	
Gender		0.324		0.050	129 (38.2)	0.316		0.035		0.637
Male	128 (37.9)		119 (35.2)		148 (34.7)		121 (35.8)		55 (16.3)	
Female	147 (34.4)		122 (28.6)		277 (36.2)		185 (43.3)		75 (17.6)	
Age (years)		<0.001		<0.001		<0.001		<0.001		0.009
60–70	43 (16.3)		38 (14.4)		46 (17.5)		77 (29.3)		30 (11.4)	
70–80	64 (31.2)		58 (28.3)		67 (32.7)		88 (42.9)		38 (18.5)	
>80	168 (56.6)		145 (48.8)		164 (55.2)		141 (47.5)		62 (20.9)	
Spouse		<0.001		<0.001		<0.001		0.538		0.097
Yes	132 (26.9)		109 (22.2)		129 (26.3)		192 (39.2)		75 (15.3)	
No	143 (52.0)		132 (48.0)		148 (53.8)		114 (41.5)		55 (20.0)	
Education		<0.001		0.001		<0.001		0.1661		0.369
Illiteracy	102 (45.7)		89 (39.9)		102 (45.7)		103 (46.2)		46 (20.6)	
Primary school	91 (37.3)		82 (33.6)		93 (38.1)		93 (38.1)		38 (15.6)	
Junior high school	41 (27.0)		36 (23.7)		43 (28.3)		57 (37.5)		22 (14.5)	
High school and above	41 (28.1)		34 (23.3)		39 (26.7)		53 (36.3)		24 (16.4)	
Financial resources		<0.001		<0.001		<0.001		<0.001		0.008
Pension	135 (32.3)		123 (29.4)		139 (33.3)		153 (36.6)		69 (16.5)	
Support from family and friends	96 (50.8)		83 (43.9)		96 (50.8)		101 (53.4)		44 (23.3)	
Others	44 (27.8)		35 (22.2)		42 (26.6)		52 (32.9)		17 (10.8)	
Current living status		<0.001		<0.001		<0.001		0.812		0.049
Live alone	79 (43.9)		63 (35.0)		78 (43.3)		72 (40.0)		29 (16.1)	
Live with family	118 (26.0)		95 (21.0)		113 (24.9)		178 (39.3)		69 (15.2)	
Others	78 (59.1)		83 (62.9)		86 (65.2)		56 (42.4)		32 (24.2)	
Monthly income (￥)		0.015		0.020		0.004		<0.001		0.100
<1,000	98 (37.1)		79 (29.9)		97 (36.7)		132 (50.0)		55 (20.8)	
1,001–3,000	55 (27.8)		52 (26.3)		55 (27.8)		63 (31.8)		29 (14.6)	
3,001–5,000	84 (43.3)		78 (40.2)		88 (45.4)		72 (37.1)		25 (12.9)	
>5,001	38 (34.9)		32 (29.4)		37 (33.9)		39 (35.8)		21 (19.2)	
Number of chronic diseases		<0.001		<0.001		<0.001		<0.001		0.016
1	37 (21.5)		35 (20.3)		39 (22.7)		39 (22.7)		18 (10.5)	
2	65 (28.9)		53 (23.6)		61 (27.1)		79 (35.1)		37 (16.4)	
≥3	173 (47.0)		153 (41.6)		177 (48.1)		188 (51.1)		75 (20.4)	
Smoking		0.099		0.464		0.707		0.843		0.520
Yes	189 (34.2)		170 (30.7)		198 (35.8)		220 (39.8)		91 (16.5)	
No	86 (40.6)		71 (33.5)		79 (37.3)		86 (40.6)		39 (18.4)	
Alcohol		0.021		0.017		0.010		0.199		0.079
Yes	223 (38.2)		197 (33.7)		226 (38.7)		241 (41.3)		107 (18.3)	
No	52 (28.7)		44 (24.3)		52 (28.2)		65 (35.9)		23 (12.7)	
Sleep schedule (h)		0.248		0.169		0.058		0.041		<0.001
<6	91 (37.0)		76 (30.9)		83 (33.7)		113 (45.9)		52 (21.1)	
6–8	118 (33.1)		104 (29.2)		122 (34.3)		127 (35.7)		40 (11.2)	
>8	66 (40.5)		61 (37.4)		72 (44.2)		66 (40.5)		38 (23.3)	

### 3.4 A binary logistic and OLS regression model of responses to the EQ-5D-3L

A binary logistic regression model was used to analyze the influence of each variable on the presence of the any problem response on each dimension of EQ-5D-3L in older adults with CVD. The study results show that financial resources affect the five dimensions of MO, SC, UA, PD, and AD. The percentage of female n is generally higher than that of male in all five dimensions. Age and number of chronic diseases affect the four dimensions of MO, SC, UA, and PD. The presence of or having a spouse, current living status, and monthly income affected the three dimensions of MO, SC, and UA, respectively. Education is a factor that influences both SC and UA dimensions. Sleep schedule is a factor that influences both PD and AD dimensions.

The OLS model is used to analyze the factors influencing each variable on the utility scores. The results of the study show that younger (60–70: beta = 0.106,95% CI = 0.059–0.154, 70–80: beta = 0.075,95% CI = 0.027–0.123), having a spouse (beta = 0.063,95% CI = 0.014–0.112), financial independence (pension: beta = 0.069,95% CI = 0.017–0.122, other: beta = 0.103,95% CI = 0.050–0.155), living with family (beta = 0.119,95% CI = 0.048–0.190) and number of one chronic disease (beta = 0.043,95% CI = 0.001–0.084) were protective factors affecting health utility scores. Illiteracy (beta = −0.075, 95% CI = −0.137 to −0.138) and the number of three or more chronic diseases (beta = −0.070, 95% CI = −0.111 to 0.028) are the risk factors affecting health utility. See Table 4.

**Table 4:** A binary logistic and OLS regression model of responses to the EQ-5D for total.

	Mobility		Self-care		Usual activities		Pain/discomfort		Anxiety/depression		HRQoL score	
	OR	95% CI	OR	95% CI	OR	95% CI	OR	95% CI	OR	95% CI	beta	95% CI
Age (years)												
>80	1.00(ref.)		1.00(ref.)		1.00(ref.)		1.00(ref.)		1.00(ref.)		1.00(ref.)	
60–70	0.226*	0.142,0.361	0.302*	0.185,0.493	0.291*	0.182,0.466	0.501*	0.328,0.765	0.687	0.398,1.187	0.106*	0.059,0.154
70-80	0.391*	0.255,0.601	0.482*	0.308,0.753	0.476*	0.308,0.736	0.846	0.565,1.265	1.002	0.611,1.643	0.075*	0.027,0.123
Spouse												
No	1.00(ref.)		1.00(ref.)		1.00(ref.)		1.00(ref.)		1.00(ref.)		1.00(ref.)	
Yes	0.519*	0.341,0.791	0.440*	0.284,0.682	0.464*	0.303,0.710	1.157	0.776,1.724	0.885	0.544,1.439	0.063*	0.014,0.112
Education												
High school and above	1.00(ref.)		1.00(ref.)		1.00(ref.)		1.00(ref.)		1.00(ref.)		1.00(ref.)	
Illiteracy	1.778	0.952,3.317	2.113*	1.095,4.078	2.116*	1.120,4.001	1.006	0.574,1.763	0.905	0.446,1.834	−0.075*	−0.137,-0.138
Primary school	1.506	0.850,2.667	1.669	0.915,3.043	1.738	0.971,3.112	0.843	0.508,1.399	0.822	0.431,1.570	−0.025	−0.080,0.030
Junior high school	1.158	0.639,2.099	1.187	0.635,2.218	1.319	0.724,2.403	1.240	0.744,2.068	1.011	0.518,1.974	−0.006	0.053,0.042
Financial resources												
Support from family and friends	1.00(ref.)		1.00(ref.)		1.00(ref.)		1.00(ref.)		1.00(ref.)		1.00(ref.)	
Pension	0.423*	0.265,0.674	0.562*	0.347,0.909	0.483*	0.301,0.774	0.609*	0.400,0.929	0.885	0.529,1.483	0.069*	0.017,0.122
Others	0.433*	0.251,0.746	0.384*	0.212,0.695	0.391*	0.222,0.687	0.431*	0.265,0.701	0.393*	0.206,0.750	0.103*	0.050,0.155
Current living status												
Others	1.00(ref.)		1.00(ref.)		1.00(ref.)		1.00(ref.)		1.00(ref.)		1.00(ref.)	
Live alone	0.596	0.347,1.024	0.260*	0.148,0.456	0.412*	0.237,0.714	1.203	0.718,2.015	0.683	0.367,1.269	0.104*	0.032,0.176
Live with family	0.524*	0.317,0.866	0.274*	0.164,0.459	0.352*	0.211,0.587	1.292	0.801,2.083	0.679	0.384,1.203	0.119*	0.048,0.190
Monthly income (￥)												
>5,001	1.00(ref.)		1.00(ref.)		1.00(ref.)		1.00(ref.)		1.00(ref.)		1.00(ref.)	
<1,000	0.438*	0.231,0.832	0.384*	0.196,0.754	0.417*	0.218,0.798	1.839*	1.031,3.281	1.148	0.560,2.354	0.043	−0.021,0.109
1,001–3,000	0.480*	0.258,0.890	0.556	0.292,1.057	0.458*	0.244,0.859	0.935	0.537,1.629	0.792	0.397,1.580	0.034	−0.029,0.097
3,001–5,000	1.661	0.934,2.955	1.874*	1.031,3.407	1.802*	1.009,3.222	1.020	0.601,1.732	0.644	0.327,1.266	−0.042	−0.06,0.127
Number of chronic diseases												
2	1.00(ref.)		1.00(ref.)		1.00(ref.)		1.00(ref.)		1.00(ref.)		1.00(ref.)	
1	0.794	0.468,1.348	0.980	0.561,1.711	0.882	0.516,1.507	0.594*	0.371,0.950	0.654	0.349,1.224	0.043*	0.001,0.084
≥3	2.056*	1.374,3.074	2.176*	1.419,3.338	2.386*	1.580,3.602	1.879*	1.308,2.698	1.188	0.750,1.882	−0.070*	−0.111,0.028
Alcohol												
No	1.00(ref.)		1.00(ref.)		1.00(ref.)		1.00(ref.)		1.00(ref.)		1.00(ref.)	
Yes	0.956	0.622,1.468	0.950	0.606,1.489	0.915	0.594,1.410	1.004	0.684,1.473	0.815	0.484,1.374	0.017	−0.019,0.052
Sleep schedule (h)												
<6	1.00(ref.)		1.00(ref.)		1.00(ref.)		1.00(ref.)		1.00(ref.)		1.00(ref.)	
6–8	0.068	0.455,1.022	0.702	0.459,1.074	0.839	0.556,1.265	0.694*	0.484,0.995	0.480*	0.299,0.769	0.033	−0.004,0.070
>8	0.877	0.537,1.432	0.962	0.579,1.598	1.286	0.784,2.109	0.716	0.457,1.121	0.954	0.568,1.604	−0.036	−0.090,0.018

*p < 0.05.

## 4 Discussion

To the best of our knowledge, this was the first study to evaluate the health quality of life and its associated factors among older adults with cardiovascular disease in eastern China. EQ-5D instrument has been widely used in pharmacoeconomic analysis and the evaluation of health policy program as a consistent and transparent measurement of the output of health care and public health system (28). Further, our study identified factors associated with HRQoL that could be targeted by interventions to improve patients’ HRQoL and reduce burden of the disease.

Our findings demonstrated that CVD diagnosis was significantly associated with impaired HRQoL. The study results show that this target population scores 0.774, which is much lower than the HRQoL in the general population (0.959) (29). Aya Barham et al. (30) scored 0.62 on the EQ-5D for people with cardiovascular disease (one type of disease) in Nablus, Palestine, which is much lower than the results of this study. These differences in utility values could be due to variations in patients’ profiles, sociocultural beliefs, and types of CVD across study settings as well as access to medical care and differences in EQ-5D-3L tariffs utilized.

Among older adults with CVD, females report slightly higher scores than males. This differs from the Korean study of the older adults, which found lower scores for females than for male (31). This may be due to the different study subjects, with the Korean study targeting all older adults and the current research focusing on older adults with CVD. In addition to this, compared to Korea, female respondents were younger than males in the estimation of the relative factors in this study, which may have resulted in the gender difference in this study being offset by the age difference. This may also be related to the level of education, which was significantly higher in the present study population than in Korea, and women were more educated than men, and sick women with higher education were more aware of healthy lifestyles, which had an impact on health-related quality of life.

The relationship between sleep and HRQOL has been studied. Short sleep may impair cognitive function, increase fatigue, and increase the risk of chronic diseases such as diabetes, obesity, and hypertension, leading to a poorer quality of life (32). In addition to this, the current study also finds an effect of sleep schedule on PD and AD, and there is no detailed study in Chen-Wei Pan and Sujeong Mun’s et al. (31) analysis showing the effect of sleep schedule on the dimensions of the scale, which is a relative improvement in the present study.

The results of the study show that age and number of chronic diseases were the key factors affecting the HRQoL score and the four dimensions of MO, SC, UA, and PD. This is similar to previous studies in that older adults have longer disease duration, the decline of HRQoL with older individuals could be attributed to increased deterioration of physical functioning, which might increase CVD progression and non-cardiac comorbidities leading to reduced overall HRQoL (33, 34). Financially independent (pension and other sources of income) older adults with CVD have relatively higher health indices. These are similar to existing research findings (21, 35). Financial independence also had a positive effect on five dimensions of EQ-5D-3L, which is similar to the findings of Li et al. (36). It can be assumed that differences in living environments, according to socioeconomic status, cause gaps among social classes in the quality of information available and the healthcare system. Therefore, if customized patient interventions are developed considering differences in financial independence, patients’ HRQOL may be improved.

We observe that the reduction in the HRQoL scores is strongly associated with having problems in the pain dimension (PD) in patients with CVD. These findings were in line with previous studies in other regions (21, 37, 38). In a study in Thailand, it was found that 50% of people with diabetes had PD problems (39). It has also been noted that the intensity of pain can affect HRQoL (37). Therefore, there is a need to focus on pain in the older adults caused by cardiovascular disease and discuss various pain reduction methods with clinical staff. This study utilizes different sociodemographic characteristics of EQ-5D-3L to provide a reference for HRQoL scores of Chinese older adults with CVD. Those results are essential in informing policies to improve CVD treatment and health outcomes and promote regional and interstate planning of CVD services.

Moreover, in the older adults, to overcome the ceiling effect and some other characteristics of the EQ-5D data, different regression models are applied to ensure the validity and robustness of the estimates when exploring the relationship between CVD and HRQoL. Although the study results show that the results of the OLS model were more accurate, further research is needed to test different populations using other methods.

## 5 Limitation

There are some limitations to this study. First, since our study was a cross-sectional survey, it is not possible to demonstrate causal relationships between associated factors and HRQoL. Second, patients were recruited from the eastern part of the country, and thus, our conclusions cannot be generalized to CVD patients in Chinese. It is suggested that future studies should consider expanding the sample to include more geographic areas in order to gain a more comprehensive understanding of the impact of economic factors on health-related quality of life (HRQoL) in older patients with cardiovascular disease. Thirdly, there is no specific EQ-5D norm for the oldest-old Chinese population, thus we adopted the TTO value set for the overall Chinese population. At the same time EQ-5D-3L as a simplified version of EQ-5D-5L, the limitations of specific own application defects. Further studies could be dedicated to develop the EQ-5D norm for this population to better understand their self-reported quality of life.

## 6 Conclusion

We find that the HRQoL of Chinese older adults with CVD is not high (0.774). Age, number of chronic diseases, financial independence, and sleep schedule all affected HRQoL, and the PD dimension of the EQ-5D-3L has a significant effect on HRQoL scores. Therefore, future intervention efforts aimed at improving HRQoL in CVD patients should be designed with a focus on modifiable factors such as controlling progression of CVD and revention, treatment of chronic diseases and improving sleep quality. PD relief can be explored with clinical professionals for patients with pain problems. These inform the provision of targeted services for older adults with CVD. Further research is needed to determine whether these estimates are consistent across different types of older adults with CVD or other diseases.

## Data availability statement

The raw data supporting the conclusions of this article will be made available by the authors, without undue reservation.

## Ethics statement

The studies involving humans were approved by this study was consistent with “Zhengzhou University Life Science Ethics Committee” [Ethics approval code ZZUIRB2022-07]. Participants were required to provide informed consent. The studies were conducted in accordance with the local legislation and institutional requirements. The participants provided their written informed consent to participate in this study.

## Author contributions

LW: Data curation, Writing – original draft, Writing – review & editing. GY: Conceptualization, Data curation, Methodology, Writing – original draft. HD: Formal analysis, Methodology, Writing – original draft. XL: Methodology, Project administration, Writing – original draft. YH: Funding acquisition, Writing – review & editing.
