# Supplementary material for: Impact of cardiovascular disease on health-related quality of life among older adults in eastern China: evidence from a national cross-sectional survey
Source: Front Public Health. 2024 Jan 15;11:1300404. doi: 10.3389/fpubh.2023.1300404 (PMC10822954; doi:10.3389/fpubh.2023.1300404)
Supplement: Supplementary file 1 [file Table_1.docx]

Table A1 Tobit regression analysis for HRQoL score

| Variables | HRQoL score | |
| --- | --- | --- |
|  | *Coef.* | *95% CI* |
| **Age(years)** |  |  |
| >80 | 1.00(ref.) | |
| 60-70 | 0.106* | 0.061,0.152 |
| 70-80 | 0.075* | 0.031,0.120 |
| **Spouse** |  |  |
| No | 1.00(ref.) | |
| Yes | 0.063* | 0.019,0.107 |
| **Education** |  |  |
| High school and above | 1.00(ref.) | |
| [Illiteracy](javascript:;) | -0.075* | -0.135,-0.016 |
| Primary school | -0.025 | -0.078,0.029 |
| Junior high school | -0.006 | 0.060,0.049 |
| **Financial resources** |  |  |
| support from family and friends | 1.00(ref.) | |
| Pension | 0.069* | 0.023,0.116 |
| Others | 0.103* | 0.050,0.155 |
| **Current living status** |  |  |
| Others | 1.00(ref.) | |
| Live alone | 0.104* | 0.047,0.160 |
| Live with family | 0.119* | 0.067,0.172 |
| **Monthly income(￥)** |  |  |
| >5001 | 1.00(ref.) | |
| <1000 | 0.043 | -0.018,0.106 |
| 1001-3000 | 0.034 | -0.025,0.093 |
| 3001-5000 | -0.042 | -0.099,0.015 |
| **Number of chronic diseases** |  |  |
| 2 | 1.00(ref.) | |
| 1 | 0.043* | -0.005,0.091 |
| ≥3 | -0.070* | -0.109,0.030 |
| **Alcohol** |  |  |
| No | 1.00(ref.) | |
| Yes | 0.017 | -0.024,0.058 |
| **Sleep schedule(h)** |  |  |
| <6 | 1.00(ref.) | |
| 6-8 | 0.033 | -0.006,0.072 |
| >8 | -0.036 | -0.085,0.012 |

**P*<0.05
